# Temporal Trends in HIV-1 Subtypes and Antiretroviral Drug Resistance Mutations in Istanbul, Türkiye (2021–2024): A Next-Generation Sequencing Study

**DOI:** 10.3390/v17040478

**Published:** 2025-03-27

**Authors:** Murat Yaman, Begüm Saran Gülcen, Kübra Özgüler, Muammer Osman Köksal, Serap Demir Tekol, Arzu İlki

**Affiliations:** 1 Medical Microbiology, Marmara University Pendik Research and Training Hospital, Istanbul 34899, Türkiye; ailki@marmara.edu.tr; 2 Medical Microbiology, Fatih Sultan Mehmet Research and Training Hospital, Istanbul 34752, Türkiye; begumsaran@gmail.com; 3 Medical Microbiology, Kartal Dr. Lutfi Kirdar City Hospital, Istanbul 34865, Türkiye; kubraozguler@hotmail.com (K.Ö.); serapdemir@yahoo.com (S.D.T.); 4 Department of Medical Microbiology, Istanbul Faculty of Medicine, Istanbul University, Istanbul 34093, Türkiye; muammerosmankoksal@istanbul.edu.tr; 5 Department of Medical Microbiology, Faculty of Medicine, Marmara University, Istanbul 3484, Türkiye

**Keywords:** human immunodeficiency virus, HIV-1 mutations and subtypes, next-generation sequencing, antiretroviral drug resistance

## Abstract

HIV-1 genotyping and drug resistance tests are routinely performed in virology laboratories in some countries, aiding clinical management. In Istanbul, between January 2021 and March 2024, plasma samples from 1029 HIV-1-infected patients were analyzed using the NGS method, and mutation and drug resistance results were retrospectively evaluated alongside demographic data. Subtype B (54.4%) was most frequent in Turkish patients, while Subtype A1 (43.5%) was predominant among foreign nationals. The most common CRFs were CRF02_AG (3.8%) and CRF56_cpx (1.6%). According to the change in detection rates during the study period, Subtype B decreased, and Subtype A increased. The most frequent mutations detected were A62V (38.7%) and M184V (22.4%) for NRTIs; E138A (55.5%) and E138G (11.5%) for NNRTIs; M46I (33.3%) and M46L (25%) for PIs; and E92Q and G for INIs (total rate: 35.2%). Darunavir/ritonavir had the highest sensitivity rate, while resistance rates for NNRTIs and INIs increased over time. We anticipate that this study, in which we evaluate the routine use of an FDA-approved NGS kit alongside integrated bioinformatics data analysis and automated reporting software for the first time in Türkiye, will contribute to both national and international molecular epidemiological data and public health strategies by providing reliable results that align with international standarts.

## 1. Introduction

HIV-1 comprises four primary groups: M, N, O, and P [1,2]. Group M, the predominant group globally accountable for the HIV-1 pandemic, is subdivided into 10 subtypes (A–D, F–H, and J–L) and 9 sub-subtypes (A1–A4, A6–A8, F1 and F2) [3,4,5,6]. The genetic diversity of HIV arises from mutations resulting from the high mistake rate of viral RNA polymerases, which lack a proofreading mechanism during the fast replication cycle, as well as from recombinations between diploid genomes [7,8]. The circulating recombinant forms (CRFs), which have emerged among Group M subtypes, are reported to have reached a total of 158 in the current database [9,10]. The heterogeneous and dynamic distribution of HIV-1 subtypes and recombinant forms in different geographic regions contributes significantly to the genetic diversity of the HIV-1 pandemic [10,11,12,13].

The global molecular epidemiology of HIV-1 varies across time, countries, and even regions within countries where population density varies. According to WHO European Region HIV/AIDS surveillance data, the rate of HIV diagnoses increased by 30.8% between 2021 and 2022, with 79,144 (71.6%) of the 110,486 HIV diagnoses reported across Europe being reported from Eastern Europe [14]. According to WHO/UNAIDS (The Joint United Nations Programme on HIV/AIDS) data, between 2010 and 2020, HIV infections decreased by 23%, with a large part of 38% in Eastern and Southern Africa, while they increased by 21% in Latin America, 22% in the Middle East and North Africa, and 72% in Eastern Europe and Central Asia [15].

The knowledge of the diversity of HIV-1 subtypes is important for the effectiveness of diagnostic tests and viral load analyses, the detection and monitoring of drug-resistant mutations (DRMs), and the development and testing of vaccines. It has been reported that certain subtypes increase the risk of virological failure; differences in the patterns of DRMs among HIV-1 subtypes and their impact on clinical outcomes are still being investigated [16,17].

The HIV replication cycle offers multiple potential targets for genetic interruptions of infection. The mostly target of antiretroviral therapy (ART) regimens for HIV-1 is the enzymes involved in the HIV-1 replication cycle. The main classes of ART include nucleoside reverse transcriptase inhibitors (NRTIs), non-nucleoside reverse transcriptase inhibitors (NNRTIs), protease inhibitors (PIs), and integrase inhibitors (INIs) [18]. Mutational differences in the replication stages cause HIV-1 drug resistance [19]. The emergence of HIV drug-resistant (HIVDR) variants can be triggered by selective pressure from drugs [8]. Combination therapy, which targets multiple steps in viral replication, usually delays selection of HIV mutants and prevents emergence of drug-resistant mutations [20].

HIV-1 drug resistance poses a potential global threat to the long-term success of ART. The World Health Organization (WHO), the U.S. International Antiviral Society, and the European AIDS Clinical Society (EACS) have reported that HIV-1 drug resistance testing (DRT) in individuals infected with HIV-1 would be beneficial in guiding the initiation or modification of ART [21,22,23].

HIV-1 DRT can be conducted through phenotypic and genotypic analyses. Recombinant phenotypic assays have been developed and used for HIV [16]. Phenotypic analyses are rarely performed today due to the intensive laboratory workload they require, their relatively high cost, difficulty in standardization, and delays in reporting results [18,24,25].

HIV-1 DRT using genotypic methods is based on sequence analyses of the pol region of HIV-1 group M, allowing for the identification of subtypes; additionally, phylogenetic analyses and molecular epidemiological assessments can also be conducted [6,26,27,28,29].

Genotypic HIV DRT involves reverse transcriptase PCR (RT-PCR) amplification of regions of the polymerase and protease genes and sometimes the integrase gene, followed by nucleotide sequencing of the amplicons. Sanger sequencing in sequence-based genotypic HIV-1 DRT has significant disadvantages due to variable test performance, challenges in standardizing the testing process, limited detection capabilities for HIV-1 drug resistance mutations (DRMs), and consequently, incomplete reporting of results [30,31,32].

Several FDA-approved sequencing systems are commercially available to detect HIV-1 DRMs. These include the TruGene system (Bayer HealthCare, Berkeley, CA, USA), approved in 2005, and the ViroSeq system (Celera Diagnostics, Alameda, CA, USA), approved in 2008 [33]. However, the ViroSeq system was discontinued at the end of 2021. The Sentosa^®^ SQ HIV Genotyping Assay (Vela Diagnostics USA, Inc., Fairfield, NJ, USA) received approval for in vitro use from the US Food and Drug Administration (FDA) in 2019 for the detection of HIV-1 DRMs in the genome using the Next Generation Sequencing (NGS) method [34,35]. This test is used as an adjunct to the therapeutic management of patients diagnosed with HIV-1 Group M infection with viral loads of at least 1000 RNA copies per mL in EDTA plasma specimens [35]. Interpreting HIV-1 DRT is complex and involves dynamic processes that require regular monitoring of up-to-date data and algorithms. In addition to the updated Stanford interpretation algorithm integrated into the workflows of the WHO’s HIVDR database, analyses can also be performed automatically using France’s ANRS and Belgium’s Rega databases with the Sentosa^®^ SQ Suite software, and reporting is carried out using the Sentosa^®^ SQ Reporter server [34,36,37].

The disruptions in the delivery of healthcare services during the COVID-19 pandemic have increased deviations in the implementation of the WHO’s global health strategies aimed to end the HIV epidemic by 2030 [38,39]. As a result, previous gains in the fight against HIV have regressed, and HIV continues to pose a global public health threat.

We hypothesize that HIV-1 drug resistance trends in Istanbul are influenced by demographic shifts and changes in ART regimens.

In Türkiye, the total number of persons who tested positive for HIV-1, as confirmed by testing from 1985 to 2014, was 8673; however, the most recent national statistics (as of 7 November 2024) indicate this figure has risen to 45,835 [40,41]. The number of HIV-1 positive individuals detected in the last ten years has increased by more than five times, which is concerning. Türkiye is a geostrategic crossroads where migrants from many countries come for various reasons and blend together. In our study, we aimed to make a meaningful contribution to current epidemiological data by analyzing HIV-1 subtypes, recombinant forms, drug resistance profiles, and mutations in Turkish and migrant cases using the FDA-approved next-generation sequencing method.

## 2. Materials and Methods

### 2.1. Study Population and Sample Collection

In this study, routine HIV-1 genotyping and drug resistance testing performed using the NGS method at the ISLAB_1 central laboratory of Kartal Dr. Lütfü Kırdar City Hospital in Istanbul between January 2021 and March 2024 were retrospectively evaluated. A total of 1029 HIV-1 infected patients, aged 0 to 79 years, were included in the analysis; these comprised individuals who were either treatment-naive or assumed to have exhibited virological failure during antiretroviral therapy.

The analysis results were obtained from the Laboratory Information System (LIS) archives, while patients demographic data (age, ethnicity, and year) were sourced from the hospital information management system database. In our study, data outside the specified date range and duplicate reports were excluded from the analysis.

EDTA plasma samples collected from patients for HIV-1 genotypic resistance testing were aliquoted upon arrival and stored at −80 °C until tested by NGS. Patients with a viral load of >1000 copies/mL were included in the HIV-1 genotypic drug resistance test by checking via the LIS.

### 2.2. HIV-1 RNA Detection and Viral Quantification

HIV-1 RNA extraction was performed on the QIAsymphony SP instrument (Hilden, Germany) using the QIAsymphony DSP Virus/Pathogen Midi Kit (Qiagen, Hilden, Germany) from patients’ plasma (EDTA) samples (1000 µL), and 60 µL of eluate was obtained. Twenty microliters of the extracted eluate were processed for HIV-1 RNA detection and quantification using the artus^®^ HI Virus-1 RG RT-PCR kit (Qiagen, Germany) in accordance with the manufacturer’s instructions on the Rotor-Gene^®^ Q device (Qiagen, Germany) (LOD: 76.4 IU/mL−34.4 copies/mL) [42].

### 2.3. Next Generation Sequencing

**Figure 1:**
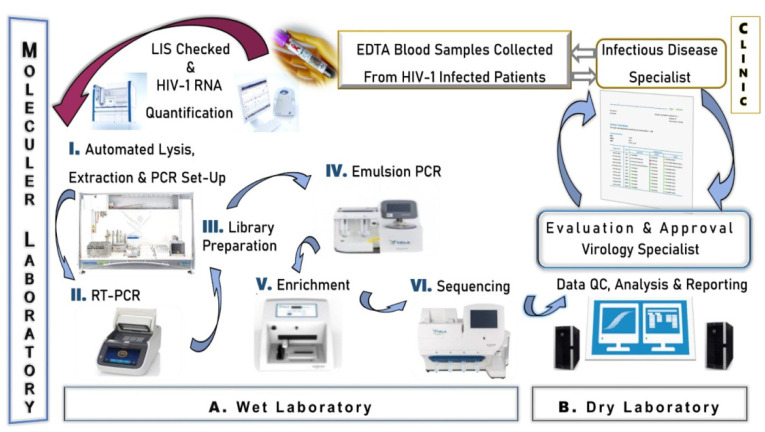
Workflow of Next Generation Sequencing.

The Sentosa HIV-1 genotyping assay (Vela Diagnostics, Hamburg, Germany) was used according to the manufacturer’s instructions [35]. The Sentosa HIV-1 genotyping assay is a NGS system that has an internationally valid FDA certificate for routine testing use, can detect all three drug resistances simultaneously, and has automatic and objective result output with the integration of bioinformatics and detailed result reporting processes into the test work system. This platform provides an NGS-based integrated workflow intended for detection and identification of drug resistance-associated HIV-1 genotypic mutations in protease (PR), reverse transcriptase (RT), and integrase (IN) genes. In each study, the assay allows the analysis up to 15 clinical samples simultaneously accompanied by a positive control. The NGS workflow (Figure 1) systematically illustrates the processes carried out in both the wet and dry laboratory areas of the molecular laboratory unit.

#### 2.3.1. Wet Laboratory Section

I. RNA Extraction. Approximately 1 mL of patient plasma samples were collected in 1.5 mL tubes (Eppendorf, Hamburg, Germany) and vortexed separately for 30 s. Afterwards, 730 µL of supernatant was transferred to new tubes (Eppendorf, Germany). Nucleic acid extraction was performed using the Virus Total Nucleic Acid Plus II Kit (Vela Diagnostics, Hamburg, Germany) in a robotic system integrated into the Sentosa SX101 (Vela Diagnostics, Hamburg, Germany) instrument. This process took approximately 2 h and 10 min, resulting in 60 µL of RNA eluate.

II. PCR Amplification. RT-PCR was performed using the Sentosa SQ HIV Reagents (Vela Diagnostics, Hamburg, Germany) kit, which first involved reverse transcriptase (RT) and then PCR. The test was performed with a single thermal cycling program in approximately 4 h and 30 min using four primer pools to amplify the RT, PR, and IN genes.

The amplification process was initiated with a single cycle of denaturation at 94 °C for 2 min. Following this, a total of 50 cycles were performed under the following temperature and duration conditions: 15 s at 94 °C, 15 s at 57 °C, 15 s at 56 °C, 15 s at 55 °C, 15 s at 54 °C, and 15 s at 53 °C. Finally, an extension step was conducted at 68 °C for 2 min. All of these procedures were carried out using a thermal cycler (Applied Biosystems, Foster City, CA, USA SimpleAmp).

III. Library Preparation. A library was created from amplicons of HIV-1 RT, PR, and IN genes using the Sentosa SQ HIV Genotyping Solutions kit on the Sentosa SX101 (Vela Diagnostics, Hamburg, Germany). The library preparation process involved adding adapters to identify the DNA molecule before sequencing. This process was completed in approximately 4 h and 15 min.

IV. Emulsion PCR. The emulsion PCR (em-PCR) process, in which the individual DNA molecule in each microdroplet is clonally amplified, was performed using the Sentosa ST Template Kit (Vela Diagnostics, Hamburg, Germany) on the Sentosa ST401 device. This process was completed in approximately 5 h and 30 min.

V. Enrichment. The enrichment process of amplicons/DNA fragments obtained through emulsion PCR (em-PCR) prior to sequencing was performed using the Sentosa ST Template Kit on the Sentosa ST401 instrument (Vela Diagnostics, Hamburg, Germany). This process was completed in approximately 45 min.

VI. Sequencing. Sequencing was performed using the Sentosa SQ301 device (Vela Diagnostics, Hamburg, Germany) with the Sentosa SQ Sequencing kit and chip (Vela Diagnostics, Hamburg, Germany), employing semiconductor sequencing technology. The HXB2 sequence was used as a reference for the HIV-1 RNA sequence analysis. This process was completed in approximately 4 h and 15 min.

#### 2.3.2. Dry Laboratory Section

Reporting & Test Approval. Stanford University, HIV database; (HIVdb, v.8.2), the Agence Nationale de Recherches sur le Sida (ANRS, v.2016.26), and the Rega Institute (v.9.1) algorithms were used to identify clinically significant drug-resistant mutations. Analyses were performed based on RT (NRTI, NNRTI), PR, and IN inhibitors mutations and combinations of these mutations that produce different levels of HIVDR. Variants including HIV-1 subtypes and CRFs were also included in these analyses. With the integrated operation of Sentosa SQ Suite and SQ Reporter (v2.5.0005, Vela Diagnostics, Hamburg, Germany) software, the automatic process for the analysis of sequence data and the conversion of results into report format was completed in approximately 4 h.

The Vela NGS system includes automated data analysis for subtype reporting. The determination of HIV subtypes in the Sentosa SQ Genotyping test is based on sequence information obtained from the target region, including Protease, Reverse Transcriptase (RT), and Integrase. HIV-1 subtyping is performed within Group M, ranging from A to K4. This process is conducted through automated phylogenetic tree analysis integrated into the SQ reporting system.

With an advanced molecular test that we provide as a routine service to many hospitals in Istanbul, the identified drug resistance mutations, the potential effect of each mutation on the effectiveness of the relevant drug, HIV subtypes, and variant information were analyzed in detail. Under the supervision of a virology specialist, HIV resistance test analyses were meticulously evaluated for each patient, consultations with the requesting clinical unit were conducted when necessary, and the reports were approved through the LIS.

### 2.4. Ethics Statement

This retrospective and cross-sectional study was approved (Approval number: 09.2024.223) by the Clinical Research Ethics Committee of the Marmara University School of Medicine and was conducted in accordance with the principles of the Declaration of Helsinki. All data were processed in a pseudonymized form.

### 2.5. Statistical Analysis

In our study, all retrospective data obtained from the laboratory information management system were recorded with Microsoft Excel version 16 (Microsoft Corp., Redmond, WA, USA). A statistical analysis was performed with the IBM SPSS Statistics 27.0 package for Windows (SPSS Inc., Chicago, IL, USA) software and GraphPad Prism (GraphPad version 10.0, San Diego, CA, USA). Continuous variables were presented as median and interquartile range (IQR), while categorical variables were presented as frequency and percentage (%). The Kolmogorov-Smirnov test was used to evaluate the conformity of the data to normal distribution. For continuous variables that did not show normal distribution, the Mann-Whitney U test was used to compare binary groups. The Pearson chi-square test, or Fisher’s exact test, when appropriate, was used to compare categorical data. Fleiss’s Kappa test was applied to evaluate the level of agreement among more than two raters. A *p*-value of 0.05 or less was considered statistically significant. For the Fleiss Kappa, the following benchmark was adopted: <0.40 poor, 0.40–0.75 intermediate to good, and >0.75 excellent [43].

## 3. Results

This study conducted a retrospective analysis of the relationship between viral load, subtypes, and drug resistance, incorporating demographic data from 1029 HIV-1 infected patients who underwent HIV-1 genotyping and drug resistance assessment via the NGS method at a single center in Istanbul (ISLAB-1).

### 3.1. Demographic Characteristics of HIV-1 Positive Patients

Demographic data and mean viral loads of the patients are presented in Table 1. It was determined that 94% of the patients (967/1029) were of Turkish origin and 89.6% were male. The rate of female cases was 9.9% in Turkish and 17.7% in foreigners. The most common age group identified was 25–34 years, comprising 35.1% of Turkish cases and 43.5% of foreign cases. The median age was 36 years (IQR 22–44). The median age was found to be greater in women than in males, with 40 years for Turkish citizens and 41 years for foreigners. The overall mean HIV-1 RNA level of the samples was 178,000 copies/mL (range 39,900–674,000 copies/mL) (Table 1).

The median HIV viral load for Turkish patients was 1.72 × 10^5^ (IQR: 4.03 × 10^4^–6.8 × 10^5^) copies/mL, which was lower than that for foreigners at 2.29 × 10^5^ (IQR: 2.5 × 10^4^–5.7 × 10^5^) copies/mL; however, this difference was not statistically significant (*p* = 0.791).

### 3.2. Prevalence of HIV-1 Genotypes by Year and Demographic Data

Sufficient readings were not obtained for 98 codons in the RT region, 117 in the protease region, and 52 in the integrase region. Consequently, RT mutations were investigated in 931 (88.1%), PI mutations in 912 (86.3%), and INI mutations in 977 (92.4%) of the 1029 patients using sequence analysis.

**Figure 2:**
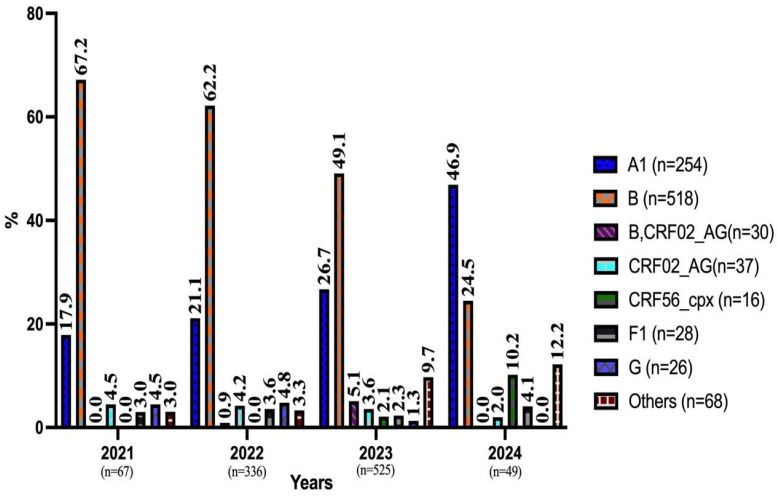
Changes in HIV-1 Subtypes Over The Years.

The distribution of HIV-1 cases (n = 977) by year, based on the highest number of reads, is shown in Figure 2. Rare subtypes with a frequency of less than 1% (C, D, J, CRF01_AE, CRF28_BF, CRF43_02G, CRF63_02A1, CRF13_cpx, and CRF14_BG) were categorized under the “Others” group. Subtype B and Subtype A1, ranked as the first and second most common subtypes, were the first two among all subtypes, respectively. In our study, as we approach 2024 (including 2024), an increase was observed in the detection rates of subtype A and Others, while a decrease was noted in the detection rates of subtype A and Others, while a decrease was noted in the detection rate of subtype B (Figure 2).

Subtype B (54.4%, 498/915) was the most frequently detected subtype in Turkish cases, and subtype A1 (43.5%, 27/62) in foreign cases. Subtype G was detected only in males. CRF56-cpx and F1 subtypes were not detected in foreign cases. Among the recombinant forms, CRF02_AG was detected in 37 cases (3.8%) and CRF56 cpx in 16 cases (1.6%), respectively. In addition, the combination of subtype B and CRF02_AG was detected in a total of 30 cases (3.1%) (Table 2).

#### 3.2.1. Prevalence of RT (NRTI, NNRTI) Resistance and Distribution of Associated Subtype Mutations

A total of 931 cases who underwent RT mutation analysis had a median age of 36 years (IQR: 28–45), and 834 (89.6%) were male. The median viral load (log) was 5.24 (IQR: 4.43–6.10).

Among NRTI-resistant, the most frequently observed mutations were A62V (38.7%) and M184V (22.4%) (Table 3). For the NNRTI-resistant cases, the two most common mutations were E138 A (55.5%) and E138 G (11.5%) (Table 3).

According to the Stanford HIV DR algorithm, multidrug resistance was identified in a total of 26 cases with high-level resistance (HLR) to drugs in the NRTI group; no cases of isolated single-drug resistance were detected. MDR was most commonly observed with resistance to Lamivudine and Emtricitabine combined (in 18 cases). CRF56_cpx was determined to be the most sensitive subtype (100%) for both NRTI and NNRTI drug groups.

In the distribution of NRTI and NNRTI drug resistance analyzed by genotypic methods, NRTI resistance mutation rate was 12.46% (116/931), while the rate of NNRTI mutations was 22.23% (207/931).

#### 3.2.2. Prevalence of PI Drug Resistance and Distribution of Associated Drug Resistance Mutations

PI mutation and drug resistance analysis was performed in 912 cases. The median age of these cases was 35 years (IQR: 28–44); 818 (89.7%) were male, and 94 (10.3%) were female.

The most frequently detected major mutations were M46 I (33.3%), M46 L (25%), and I84 V (16.6%), respectively. For accessory mutations, the most frequently were K43 T (52.9%) and Q58 E (17.6%) (Table 4).

All cases with subtypes CRF02_AG, CRF56, and F1 were found to be susceptible to PI drugs. In cases with accessory mutations, sensitivity to PI drugs, except for other than atazanavir (HLR not detected, 98.9%; 901/912), was determined to be 100%. Nelfinavir had HLR in 4 cases and was determined to be the PI drug with the lowest sensitivity (96.4%). HLR was not detected for tipranavir in any of the cases. No HLR was detected in any of the 56 foreign national cases (45 males and 11 females) evaluated for whom PI drug resistance. In the distribution of PI drug resistance analyzed by genotypic methods, the major resistance mutation rate was 1.12% (10/912), while the rate of accessory mutations was 1.75% (16/912).

#### 3.2.3. Prevalence of INI Drug Resistance and Distribution of Associated Drug Resistance Mutations

The median viral load (log) of the 977 cases in which INI mutations were investigated was found to be 5.28 (IQR: 4.61–5.87). Of these cases, 874 (89.5%) were male, and 103 (10.5%) were female. In the distribution of INI drug resistance analyzed by genotypic methods, the major resistance mutation rate was 1.12% (11/977), while the rate of accessory mutations was 4.5% (44/977).

The prevalence of major mutations varied from 5.8% to 17.6%. The most frequently observed major mutations were E92G, E92Q, and Y143C, each detected at an equal frequency of 17.6%. The two most frequently detected accessory mutations were E157Q, found in 33.3% of cases, and T97A, 29.1% (Table 5).

HLR for INI was detected in a total of 14 cases. HLR was not detected for dolutegravir, and the sensitivity of this drug (99.7%) was found to be higher than the other two drugs (94.3% for elvitegravir and 94.3% for raltegravir). All cases with B & CRF02_AG, CRF02_AG, F1, and Other subtypes were found to be sensitive to the drugs in the INI group where genomic resistance analysis was performed. Among the total of 65 foreign national cases (54 males and 11 females) in which INI resistance was evaluated, only one female case with the CRF02_AG subtype had HLR detected for both elvitegravir and raltegravir, while no HLR was detected in the other foreign cases.

The Fleiss’ Kappa (κ) test analysis results evaluating the concordance levels among the three different algorithms included in the detailed HIV-1 drug resistance report generated using the Sentosa SQ Reporter software (v2.5.0005, Vela Diagnostics, Germany) are presented in Table 6. In the comparison of algorithm-based evaluations, weak concordance (κ < 0.40) was observed for didanosine (κ = 0.236) among NRTIs and for etravirine (κ = 0.363) among NNRTIs. The concordance level among algorithmic evaluations was determined to be excellent (κ > 0.75) for lamivudine, emtricitabine, and zidovudine in the NRTI class, and for rilpivirine in the NNRTI class. We identified no drug with an excellent concordance level for the PI and INI classes. However, the concordance level was determined to be moderate to good (κ = 0.40–0.75) for indinavir/ritonavir, fosamprenavir/ritonavir, and darunavir/ritonavir in the PI class, and for elvitegravir in the INI class. In all three algorithms, the most sensitive antiretroviral medication was found to be the protease inhibitor darunavir/ritonavir.

**Figure 3:**
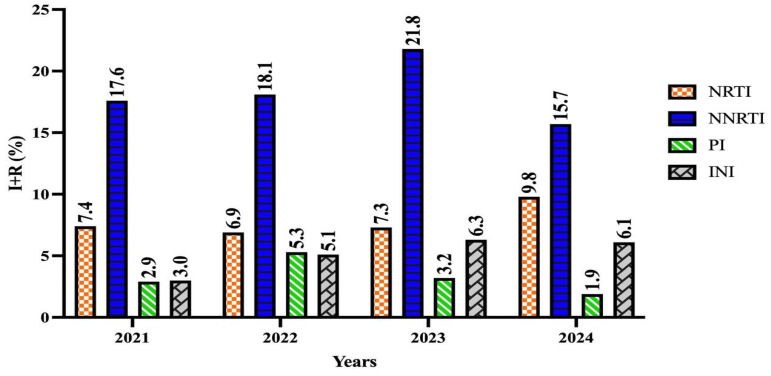
Yearly Trends in Drug Resistance Observed in HIV Treatment Drug Classes.

According to the Stanford algorithm, the rates of HIV-DR susceptibility and resistance levels (medium and high) and data between years are presented in Table 7. Over the years, the changes in HIV-DR were not found to be statistically significant (*p* > 0.05). The data reveal that the resistance rates in the NNRTI and INI groups increased over the years (Figure 3).

Detailed data of a total of nine pediatric cases (8 males and 1 female) aged 1–17 years are presented in Table 8. Multiple HIV-DR resistance was detected in a 13-year-old male case with the CRF02_AG subtype according to the Stanford algorithm. In this case, M184V and T215Y mutations for NRTI, V108I mutation for NNRTI, major I54V and V82A mutations for PI, and accessory Q95K mutation for INI (potential low-level resistance) were detected. In a female case with subtype B, M41L and M184V mutations were detected in the NRTI group and K101E mutation in the NNRTI group. HIV-1 DR was not detected in a male case of foreign origin and in three male cases aged 17, 16, and 1 years with multiple subtypes.

## 4. Discussion

The findings of this study provide valuable insights into the epidemiology of HIV-1 drug resistance and subtype distribution in Istanbul, Türkiye, which could have significant implications for treatment strategies and public health interventions. Our study revealed that the majority of the patients (94%) were of Turkish origin, with men comprising 89.6% of the cohort. The predominance of male cases aligns with global patterns, where men, particularly men who have sex with men (MSM), represent a significant proportion of HIV diagnoses in many regions [44]. The median age of the cohort (36 years) highlights the importance of targeted prevention efforts among young and middle-aged adults.

Research on HIV genetic heterogeneity has recently focused on the epidemiological and geographical changes in the distribution of HIV subtypes, particularly in relation to the movements of communities and the resulting population mixing. The ongoing refugee influx and subsequent foreign migration in Türkiye since 2015 are considered factors affecting the general population, necessitating the investigation of temporal changes in geographical patterns of subtype distribution. Subtype distribution analysis identified Subtype B as the most common among Turkish patients (54.4%), while Subtype A1 was predominant among foreign patients (43.5%). Interestingly, recombinant forms, particularly CRF02_AG, were detected in 3.8% of cases. These findings mirror global trends, where Subtype B dominates in Western regions, while recombinant forms and non-B subtypes are more prevalent in regions like Africa and Asia [44,45].

Previous studies on HIV-1 drug resistance and genetic diversity in Türkiye have revealed varying resistance profiles across different regions and patient populations [46,47,48]. Another study utilizing next-generation sequencing (NGS) identified resistance to at least one antiretroviral (ARV) drug in 22.4% (11 of 49) of the samples, with resistance rates of 6.1% for NRTIs, 12.2% for NNRTIs, 4% for PIs, and 8.1% for INIs [46]. In a study from Istanbul, significant heterogeneity in HIV-1 subtypes was observed, with Subtype B being dominant but a high prevalence of CRF02_AG also reported [47]. The drug resistance rates and subtype distributions identified in our study largely align with these previous findings. However, we observed an increasing trend in NNRTI and INI resistance over time, which may be attributed to shifts in antiretroviral therapy regimens and patient adherence. Additionally, while the overall prevalence of INI resistance mutations reported as 3.6% in a study with 169 patients, our study, which included a much larger sample size (977 patients), found an overall INI resistance prevalence of 1.1%. Similarly, the NRTI and NNRTI resistance rates in our study (12% and ~20%, respectively) were notably higher than those reported in the NGS-based study [48]. These differences may be explained by variations in sample sizes, study populations, and regional treatment practices. Given that the HIV epidemiology in Türkiye is influenced by migration patterns, it is crucial to conduct long-term and large-scale surveillance studies to monitor the evolving dynamics of drug resistance mutations at a national level.

The increasing prevalence of Subtype A1 and other rare subtypes in our cohort suggests potential cross-border transmission due to Türkiye’s geographic location at the crossroads of Europe and Asia. Similar trends have been observed in Greece and Balkan countries, where subtype diversity is associated with migratory patterns and international interactions [11,49,50]. Subtype C, which is highly prevalent in regions like Southern Africa, India and Ethiopia [51,52], was notably absent among Turkish patients, reflecting regional differences in HIV epidemiology.

Nucleoside reverse transcriptase inhibitor (NRTI) resistance was detected in 12.4% of males and 12.6% of females, with a significantly higher prevalence in foreign nationals [53] compared to Turkish patients (11.9%), The A62V and M184V mutations were the most frequently observed among NRTI-resistant cases. M184V is associated with resistance to lamivudine (3TC) and emtricitabine (FTC), which are commonly used in first-line treatment regimens, while A62V is often observed in association with NRTI resistance mutations such as Q151M, K65R, and M184V [54,55,56]. In addition, the M41L mutation stands out as the 3rd most frequently observed change. M41L usually occurs in combination with T215Y. The combination is associated with decreased sensitivity to AZT, ABC and TFV [57]. These mutations belong to the TAM type 1 pattern and are selected by thymidine analogs, a class of drugs that are no longer widely used in current therapeutic regimens [49].

Non-nucleoside reverse transcriptase inhibitor (NNRTI) resistance was observed in 22.6% of males and 18.9% of females, with E138 mutations (A/G) being the predominant mutations. Mutations at position 138 (most notably E138A) may occur as natural polymorphisms, especially in non-B subtype virus [58]. Consistent with previous reports, the present study showed a stable predominance of mutation to NNRTIs, which is partly explained by the higher prevalence of the NNRTI-associated E138A [49,58]. In vitro studies have shown that E138A confers an almost two-fold decrease in sensitivity to rilpivirine [49]. E138 mutations are followed by V179 mutations. From this mutation group, V179D is a polymorphic accessory NNRTI-selected mutation. It contributes low-level reductions in susceptibility to each of the NNRTIS. V179E is a non-polymorphic mutation occasionally selected by NVP and EFV. V179E appears similar to V179D in its effects on NNRTIs, with the same NNRTI resistance [59].

Interestingly, the prevalence of multidrug resistance (MDR) was limited to 26 cases, with the majority showing high-level resistance to both 3TC and FTC. This finding underscores the effectiveness of current treatment protocols but also highlights the need for routine resistance testing to prevent MDR emergence.

Protease inhibitor (PI) resistance was rarely observed (1.3%), the most common were M46I and M46L mutations, which is consistent with data observed in similar studies conducted recently [60,61]. M46I and M46L mutations are observed in approximately 20% and 10% of patients undergoing protease inhibitor (PI) treatment, respectively. These mutations are associated with reduced susceptibility to ATV and LPV. While M46I typically occurs either alone or in combination with mutations such as V32I, I47V, L76V, I84V, and L90M, M46L is more commonly found alone or alongside I54V and V82A [62,63]. Accessory mutations, such as K43T and Q58E, were more common but did not lead to significant resistance. In combination with other PI-resistance mutations, it may contribute to low-level PI resistance such as TPV [63,64]. These findings are consistent with global trends, where PI resistance remains relatively low due to the high genetic barrier of these drugs [60,61].

The prevalence of integrase inhibitor (INI) resistance was similarly low, with major mutations detected in only 1.7% of cases. The accessory mutation E157Q was the most frequently observed. It is almost always poorly selected in combination with other INI-resistance mutations during INI therapy. It has almost no effect against any INS alone [64]. Regimens containing the INIs bictegravir (BIC) or dolutegravir (DTG) are recommended as initial treatment for most individuals owing to their high efficacy, tolerability, safety, and high barrier to resistance; low pill burden; and low potential for drug&drug interactions [65]. However, the presence of resistance mutations, albeit rare, underscores the importance of continuous surveillance to detect emerging patterns.

Genotypic resistance interpretation systems exhibit significant discordances, even for long-established antiretroviral drugs. This is primarily due to variations in how algorithms classify mutations, which is influenced by the complexity of resistance patterns, the inclusion criteria for new mutations, and the potential impact of subtype-specific variations. For instance, the ANRS system often diverges from Rega and Stanford-HIVdb due to its inclusion of emerging mutations based on limited data [66,67]. Notably, the study showed that while there are minor variations for protease inhibitors and NNRTIs, discrepancies for NRTI resistance can reach up to 9% depending on the method used. As combination therapy becomes more prevalent globally, particularly in resource-limited settings, such differences emphasize the need for enhanced standardization and the integration of subtype-specific insights. Aligning algorithm interpretations could optimize resistance detection and support more effective implementation of WHO’s 90-90-90 goals, especially in regions like Türkiye, where genetic diversity and cross-border transmission pose additional challenges [66,67].

The findings of this study underscore the need to adapt national treatment guidelines and strengthen genotypic resistance testing as a routine practice. Ultrasensitive resistance testing demonstrates that lowering detection thresholds for pretreatment drug resistance can improve the identification of individuals at risk of virological failure, albeit at the cost of reduced specificity [68].

In our study, detailed data are presented for a total of nine pediatric cases (8 males, 1 female), aged between 1 and 17 years. Several studies conducted across different continents have reported that more than 50% of pediatric patients diagnosed with HIV-1 develop resistance, particularly to the NRTI and NNRTI groups, as part of drug resistance monitoring. For example, in a study conducted in Panama, resistant strains were detected in 54.8% of 62 pediatric patients undergoing treatment, with rates of 58.5% for the NRTI group and 39% for the NNRTI group. In addition, multiple HIV drug resistance was observed, with resistance in the combination of NRTI and NNRTI (12.2%) and in the combination of NNRTI plus PI (2.6%) [69]. In another study conducted in Kinshasa, Democratic Republic of Congo, involving a pediatric/adolescent population with a median age of 14 years, the most common resistance mutations were detected in the NNRTI (73.5%) and NRTI (61.2%) groups; furthermore, 53.1% of patients exhibited dual-class resistance (NRTI + NNRTI) [70]. Although resistance mutations appear to occur in children similarly to adults, it has been reported that virological failure may develop even more rapidly [71]. These studies underscore the prevalence of multidrug-resistant strains and highlight the importance of resistance monitoring strategies in improving treatment response. We attribute the low rate of multidrug and multiclass resistance observed during our study period (22% in two of nine cases) to the small sample size, the close follow-up of cases, and high treatment compliance.

Early detection of resistance mutations can optimize ART regimens and prevent treatment failure. Strengthened surveillance programs monitoring subtype dynamics and resistance trends are crucial, particularly in regions like Türkiye, which experience significant cross-border transmission. Adherence support programs addressing barriers, especially in populations at higher risk of resistance, and adaptation of treatment guidelines incorporating high genetic barrier drugs, such as dolutegravir, are essential to mitigate resistance risks. Routine implementation of genotypic resistance testing for all newly diagnosed patients is essential to optimize ART regimens and reduce treatment failure. National ART guidelines should prioritize INSTI-based regimens, which have demonstrated superior resistance profiles compared to NNRTI-based treatments. Multicenter studies capturing data from diverse regions within Türkiye can provide a more representative picture of resistance patterns and subtype dynamics. Targeted education and intervention programs are needed to reduce risky behaviors contributing to the spread of drug-resistant strains.

The retrospective nature of the study may introduce selection bias. Limited representation of foreign nationals and specific subpopulations may affect the generalizability of the findings. The lack of data on treatment adherence and clinical outcomes prevents a comprehensive understanding of the resistance’s clinical impact. Data on patients’ use of antiretroviral therapy (ART) and subsequent treatment failures were not included in our study. Future research should address these gaps by incorporating multicenter collaborations and long-term follow-up data.

In our study, the proportion of female participants was significantly lower than that of males. While this distribution aligns with the epidemiology of HIV in Türkiye, it presents a limitation in assessing the potential impact of sex on HIV-1 drug resistance. Previous studies have suggested that certain NNRTI mutations may be more frequently observed in females, potentially due to hormonal or immunological differences [72]. However, due to the low number of female patients in our study, it was not possible to statistically evaluate such differences. Future large-scale studies may provide a more comprehensive understanding of HIV-1 drug resistance patterns in female patients.

Our findings provide a detailed overview of HIV-1 drug resistance mutations and subtype dynamics in Istanbul. By contextualizing these results within global trends, we highlight the urgent need for robust surveillance systems, routine resistance testing, and policy shifts toward INSTI-based regimens. These measures are critical for mitigating the impact of drug resistance and advancing global efforts to control the HIV epidemic.

## 5. Conclusions

This study provides a comprehensive analysis of HIV-1 drug resistance mutations and subtype distributions among 1029 patients in Istanbul, Türkiye. The findings reveal a predominance of Subtype B among Turkish patients and Subtype A1 among foreign nationals, alongside an increasing prevalence of recombinant forms. Drug resistance mutations were identified across all major antiretroviral drug classes, with a gradual upward trend observed in resistance rates for NNRTIs and INIs over the study period. The high sensitivity of protease inhibitors, particularly darunavir/ritonavir, highlights their continued effectiveness in treatment protocols.

Our results emphasize the need for routine HIV-1 drug resistance testing as an integral component of clinical management, particularly in regions experiencing significant cross-border transmission. The use of next-generation sequencing has proven instrumental in detecting low-frequency mutations and providing a detailed molecular epidemiological perspective.

Alongside viral load, HIV subtypes, and drug resistance profiles, the individualized management of HIV antiviral therapy for both domestic and international patients significantly contributes to achieving more effective and sustainable treatment outcomes on both national and global scales.

To optimize treatment outcomes, national guidelines should prioritize integrase inhibitor (INSTI)-based regimens, given their superior resistance profiles. Strengthened surveillance programs and multicenter studies across diverse regions of Türkiye are crucial for monitoring subtype dynamics and resistance patterns. By addressing these challenges, healthcare systems can contribute significantly to achieving global HIV control goals and improving patient outcomes.

## Figures and Tables

**Table 1:** Demographic Data and Viral Load Averages of HIV-1 Positive Cases.

	All			Turkish			Others		
	All	M	F	All	M	F	All	M	F
n (%)	1029 (100)	922 (89.6)	107 (10.4)	967 (100)	871 (90.1)	96 (9.9)	62 (100)	51 (82.3)	11 (17.7)
Age Groups									
<25 n	118	109	9	114	105	9	4	4	-
25–34 n	366	341	25	339	317	22	27	24	3
35−44 n	291	257	34	273	243	30	18	14	4
45−54 n	148	128	20	137	119	18	11	9	2
>54 n	106	87	19	104	87	17	2	-	2
Age Median	36	35	41	36	35	40	34	34	41
IQR	28−44	28−44	33−49	28−44	28−44	32−48	29–43	29−42	33−53
Viral Load (log_10_)	1.78	1.84	1.03	1.72	1.81	0.98	2.29	2.39	1.50
IQR	3.99 × 10^4^– 6.74 × 10^5^	4.31 × 10^4^– 6.81 × 10^5^	1.10 × 10^4^– 5.72 × 10^5^	4.03 × 10^4^– 6.81 × 10^5^	4.36 × 10^4^– 6.84 × 10^5^	1.15 × 10^4^– 5.26 × 10^5^	2.56 × 10^4^– 5.78 × 10^5^	2.81 × 10^4^– 5.27 × 10^5^	4.61 × 10^3^– 1.12 × 10^6^

Abbreviations: IQR: Interquartile range, M: Men, F: Female, n: number of cases.

**Table 2:** Demographic Analysis of HIV-1 Subtypes by Gender and Ethnicity (n = 977).

	A1	B	B & CRF02_AG	CRF02_AG	CRF56_cpx	F1	G	Others
Gender								
Male n (%)	230 (26.3)	459 (52.5)	28 (3.2)	31 (3.5)	14 (1.6)	26 (3.0)	26 (3.0)	60 (6.9)
Female n (%)	24 (23.3)	59 (57.3)	2 (1.9)	6 (5.8)	2 (1.9)	2 (1.9)	0	8 (7.8)
Etnicity								
Turkish n (%)	227 (24.8)	498 (54.4)	28 (3.1)	33 (3.6)	16 (1.7)	28 (3.1)	23 (2.5)	62 (6.8)
Others n (%)	27 (43.5)	20 (32.3)	2 (3.2)	4 (6.5)	0	0	3 (4.8)	6 (9.7)

Abbreviations: n: number of cases.

**Table 3:** Reverse Transcriptase Associated Drug Resistance Mutations.

NRTI (n = 116)				NNRTI (n = 207)			
AA	Mutation	Prevalence n (%)	Only Mutation n (%)	AA	Mutation	Prevalence n (%)	Only Mutation n (%)
M41	L	21 (18.1)	4 (19.0)	A98	G	3 (1.4)	2 (66.6)
E44	D	6 (5.2)	6 (100)	L100	V	2 (0.9)	1 (50)
A62	V	45 (38.7)	41 (91.1)	K101	E	8 (3.8)	2 (25)
K65	R	5 (4.3)	0 (0)		Q	2 (0.9)	0 (0)
D67	R	3 (2.6)	1 (33.3)		R	8 (3.8)	7 (87.5)
	G	1 (0.8)	0 (0)	K103	E	6 (2.8)	2 (33.3)
	N	2 (1.7)	0 (0)		N	7 (3.3)	6 (85.7)
T69	D	1 (0.8)	0 (0)		R	2 (0.9)	1 (50)
	insertion	1 (0.8)	0 (0)	V108	I	11 (5.3)	10 (90.9)
K70	E	2 (1.7)	1 (50.0)	E138	A	115 (55.5)	102 (88.6)
	N	5 (4.3)	3 (60.0)		G	24 (11.5)	24 (100)
	Q	1 (0.8)	0 (0)		K	1 (0.4)	0 (0)
	R	1 (0.8)	0 (0)		Q	2 (0.9)	0 (0)
	T	2 (1.7)	0 (0)	V179	T	9 (4.3)	8 (88.8)
L74	I	1 (0.8)	0 (0)		D	14 (6.7)	10 (71.4)
V75	A	1 (0.8)	1 (100)		E	5 (2.4)	5 (100)
	M	1 (0.8)	0 (0)	Y188	C	1 (0.4)	1 (100)
Y115	F	1 (0.8)	0 (0)		L	2 (0.9)	1 (50)
M184	I	3 (2.6)	1 (33.3)	G190	S	3 (1.4)	2 (66.6)
	V	26 (22.4)	8 (30.7)	M230	I	1 (0.4)	1 (100)
L210	W	1 (0.8)	1 (100)	Y318	F	0 (0)	0 (0)
T215	C	12 (10.3)	3 (25)	N348	I	4 (1.9)	3 (75)
	D	5 (4.3)	1 (20.0)				
	E	8 (6.8)	4 (50)				
	I	1 (0.8)	0 (0)				
	S	5 (4.3)	1 (20.0)				
	Y	2 (1.7)	0 (0)				
K219	R	2 (1.7)	2 (100)				
	Q	3 (2.6)	2 (66.6)				

**Table 4:** Protease Associated Drug Resistance Mutations.

PI Major (n = 10)				PI Accessory (n = 16)			
AA	Mutation	Prevalence n (%)	Only Mutation n (%)	AA	Mutation	Prevalence n (%)	Only Mutation n (%)
D30	N	1 (8.3)	1 (100)	K20	T	2 (11.7)	2 (100)
M46	I	4 (33.3)	3 (75)	L33	F	2 (11.7)	1 (50)
	L	3 (25)	3 (100)	K43	T	9 (52.9)	8 (88.8)
I50	L	1 (8.3)	1 (100)	F53	L	1 (5.8)	1 (100)
	V	1 (8.3)	1 (100)	Q58	E	3 (17.6)	3 (100)
154	L	1 (8.3)	0 (0)	N63	D	1 (5.8)	1 (100)
	V	1 (8.3)	0 (0)				
L76	V	1 (8.3)	0 (0)				
V82	A	1 (8.3)	0 (0)				
I84	V	2 (16.6)	1 (50)				

Abbreviations: n: number of cases.

**Table 5:** Integrase Associated Drug Resistance Mutations.

Integrase Inhibitor Mutation Major (n = 11)				Integrase Inhibitor Mutation Accessory (n = 44)			
AA	Mutation	Prevalence n (%)	Only Mutation n (%)	AA	Mutation	Prevalence n (%)	Only Mutation n (%)
T66	A	2 (11.7)	2 (100)	Q95	K	2 (4.1)	1 (50)
E92	G	3 (17.6)	2 (66.6)	T97	A	14 (29.1)	12 (85.7)
	Q	3 (17.6)	3 (100)	A128	T	6 (12.5)	5 (83.3)
E138	K A	2 (11.7)	1 (50.0)	E157	Q	16 (33.3)	15 (93.7)
G140	S	2 (11.7)	0 (0)	G163	K	3 (6.2)	3 (100)
Y143	C	3 (17.6)	1 (33.3)		R	10 (20.8)	7 (70.0)
	R	1 (5.8)	0 (0)	S230	R	1(2.0)	1 (100)
S147	G	1 (5.8)	0 (0)				
Q148	H	2 (11.7)	0 (0)				
	R	1 (5.8)	0 (0)				
N155	H	1 (5.8)	0 (0)				
	T	1 (5.8)	1 (100)				
R263	K	1 (5.8)	1 (100)				

Abbreviations: n: number of cases.

**Table 6:** Evaluation Results of Drugs Used in HIV Treatment with Stanford, ANRS and Rega Algorithms and Analysis of Agreement Levels Between Raters with Fleiss’s Kappa Method.

			Methods						Statistics	
			Standford		ANRS		Rega			
			S	R (I + H)	S	R (I + H)	S	R (I + H)		
Drug Groups		Drugs	n (%)	n (%)	n (%)	n (%)	n (%)	n (%)	*p*-Value	Fleiss’s Kappa (κ)
RT (n = 931)	N R T I	ABC	890 (95.6)	41 (4.4)	889 (95.5)	42 (4.5)	919 (99)	12 (1.3)	<0.001	0.608
		AZT	886 (95.2)	45 (4.8)	898 (96.5)	33 (3.5)	899 (96.6)	32 (3.4)	<0.001	0.858
		D4T	876 (94.1)	55 (5.9)	900 (96.7)	31 (3.3)	898 (96.5)	33 (3.5)	<0.001	0.719
		DDI	874 (93.9)	57 (6.1)	924 (99.2)	7 (0.8)	903 (97)	28 (3.0)	<0.001	0.236
	N N R T I	FTC	900 (96.7)	31 (3.3)	905 (97.2)	26 (2.8)	904 (97.1)	27 (2.9)	<0.001	0.939
		3TC	899 (96.6)	32 (3.4)	904 (97.1)	27 (2.9)	903 (97)	28 (3.0)	<0.001	0.941
		TDF	904 (97.1)	27 (2.9)	922 (99)	9 (1.0)	923 (99.1)	8 (0.9)	<0.001	0.561
		EFV	850 (91.3)	81 (8.7)	907 (97.4)	24 (2.6)	909 (97.6)	22 (2.4)	<0.001	0.439
		ETR	768 (82.5)	163 (17.5)	772 (82.9)	159 (17.1)	926 (99.5)	5 (0.5)	<0.001	0.363
		NVP	844 (90.7)	87 (9.3)	906 (97.3)	25 (2.7)	909 (97.6)	22 (2.4)	<0.001	0.443
		RPV	763 (82)	168 (18)	778 (83.6)	153 (16.4)	789 (84.7)	142 (15.3)	<0.001	0.933
PI (n = 912)		ATV/r	897 (98.4)	15 (1.6)	730 (80)	182 (20)	905 (99.2)	7 (0.8)	0.294	0.020
		DRV/r	908 (99.6)	4 (0.4)	911 (99.9)	1 (0.1)	911 (99.9)	1 (0.1)	<0.001	0.499
		FPV/r	893 (97.9)	19 (2.1)	905 (99.2)	7 (0.8)	907(99.4)	5 (0.6)	<0.001	0.511
		IDV/r	897 (98.4)	15 (1.6)	901 (98.8)	11 (1.2)	905 (99.2)	7 (0.8)	<0.001	0.724
		LPV/r	897 (98.4)	15 (1.6)	897 (98.4)	15 (1.6)	909 (99.7)	3 (0.3)	<0.001	0.356
		NFV	879 (96.4)	33 (3.6)	906 (99.3)	6 (0.7)	904 (99.1)	8 (0.9)	<0.001	0.329
		SQV/r	898 (98.5)	14 (1.5)	653 (71.6)	259 (28.4)	908 (99.6)	4 (0.4)	0.002	−0.060
		TPV/r	889 (97.5)	23 (2.5)	282 (30.9)	630 (69.1)	911 (99.9)	1 (0.1)	<0.001	−0.277
INI (n= 977)		DTG	964 (98.7)	13 (1.3)	959 (98.1)	18 (1.9)	972 (99.5)	5 (0.5)	<0.001	0.297
		EVG	921 (94.3)	56 (5.7)	936 (95.8)	41 (4.2)	961 (98.4)	16 (1.6)	<0.001	0.595
		RAL	921 (94.3)	56 (5.7)	951 (97.3)	26 (2.7)	946 (96.8)	31 (3.2)	<0.001	0.383

Abbreviations: RT: Reverse Transcriptase, NRTI: Nucleoside Reverse Transcriptase Inhibitor, ABC: Abacavir, AZT: Zidovudine, D4T: Stavudine, DDI: Didanosine, NNRTI: Non-Nucleoside Reverse Transcriptase Inhibitor, FTC: Emtricitabine, 3TC: Lamivudine, TDF: Tenofovir, EFV: Efavirenz, ETR: Etravirine, NVP: Nevirapine, RPV: Rilpivirine, PI: Protease Inhibitor, ATV/r: Atazanavir/Ritonavir, DRV/r: Darunavir/Ritonavir, FPV/r: Fosamprenavir/Ritonavir, IDV/r: Indinavir/Ritonavir, LPV/r: Lopinavir/Ritonavir, NFV: Nelfinavir, SQV/r: Saquinavir/Ritonavir, TPV/r: Tipranavir/Ritonavir, INI: Integrase Inhibitor, DTG: Dolutegravir, EVG: Elvitagravir, RAL: Raltegravir, R: Resistant, I: Intermediatiate Level Resistance, H: High Level Resistance, S: Sensitive, n: Number of cases.

**Table 7:** Comparison of Susceptibilities and Resistances Detected by Stanford Algorithm in Drug Groups Used in HIV Treatment by Years.

	2021		2022		2023		2024 ^a^		*p*-Value
	S	R (I + H)	S	R (I + H)	S	R (I + H)	S	R (I + H)	
	n (%)	n (%)	n (%)	n (%)	n (%)	n (%)	n (%)	n (%)	
NRTI	63 (92.6)	5 (7.4)	299 (93.1)	22 (6.9)	455 (92.7)	36 (7.3)	46 (90.2)	5 (9.8)	0.904
NNRTI	56 (82.4)	12 (17.6)	263 (81.9)	58 (18.1)	384 (78.2)	107 (21.8)	43 (84.3)	8 (15.7)	0.464
PI	66 (97.1)	2 (2.9)	303 (94.7)	17 (5.3)	457 (96.8)	15 (3.2)	50 (98)	1 (1.9)	0.380
INI	65 (97)	2 (3)	319 (94.9)	17 (5.1)	492 (93.7)	33 (6.3)	46 (93.9)	3 (6.1)	0.674

Abbreviations: R: Resistant, I: Intermediatiate Level Resistance, H: High Level Resistance, S: Sensitive, n: Number of cases, ^a^: First three months of 2024, NRTI: Nucleoside Reverse Transcriptase Inhibitor, NNRTI: Non-Nucleoside Reverse Transcriptase Inhibitor, PI: Protease Inhibitor, INI: Integrase Inhibitor.

**Table 8:** Epidemiological and Virological Data in Pediatric Cases.

N	Gender (M/F)	Etnicity	Age	Viral Load Copy/mL	Subtype	NRTI			NNRTI			PI			INI		
						S	IR	HR	S	IR	HR	S	IR	HR	S	IR	HR
1	M	TR	13	51,220	CRF02_AG	TDF	ABC AZT D4T DDI	FTC 3TC	ETR RVP	EFV NVP	-	DRV/r	ATV/r FPV/r IDV/r LPV/r SQV/r TPV/r	NFV	DTG	EVG RAL	-
2	M	TR	15	54,814,294	B	All	-	-	All	-	-	All	-	-	All	-	-
3	M	TR	1	16,263	A1	All	-	-	All	-	-	All	-	-	All	-	-
4	M	TR	17	105,000	B + CRF02_AG + G + J	All	-	-	All	-	-	All	-	-	All	-	-
5	F	TR	2	124,854	B	AZT D4T TDF	ABC DDI	FTC 3TC	-	NVP RPV DOR EFV ETR	-	All	-	-	All	-	-
6	M	Other	17	1,040,000	B	All	-	-	All	-	-	All	-	-	All	-	-
7	M	TR	16	1,580,000	CRF02_AG	All	-	-	All	-	-	All	-	-	All	-	-
8	M	TR	16	1,100,120	CRF02_AG + G	All	-	-	All	-	-	All	-	-	All	-	-
9	M	TR	1	794,000	D + G	All	-	-	All	-	-	All	-	-	All	-	-

Abbreviations: NRTI: Nucleoside Reverse Transcriptase Inhibitor, ABC: Abacavir, DDI: Didanosine, FTC: Emtricitabine, 3TC: Lamivudine, D4T: Stavudine, TDF: Tenofovir, AZT: Zidovudine, DOR: Doravirine, NNRTI: Non-Nucleoside Reverse Transcriptase Inhibitor, EFV: Efavirenz, ETR: Etravirine, NVP: Nevirapine, RPV: Rilpivirine, PI: Protease Inhibitor, ATV/r: Atazanavir/Ritonavir, DRV/r: Darunavir/Ritonavir, FPV/r: Fosamprenavir/Ritonavir, IDV/r: Indinavir/Ritonavir, LPV/r: Lopinavir/Ritonavir, NFV: Nelfinavir, SQV/r: Saquinavir/Ritonavir, TPV/r: Tipranavir/Ritonavir, INI: Integrase Inhibitor, DTG: Dolutegravir, EVG: Elvitagravir, RAL: Raltegravir, HR: High Resistant, IR: Intermediatiate Resistance, S: Sensitive, N: Number of cases, M: Men, F: Female.

## Data Availability

Data are available within the article.
